# Voyages in Vacation, III

**Published:** 1888-10-13

**Authors:** 


### Voyages in Vacation.—III.

By One in Search of Restful Restoratives.

(*Continued from page 7.*)

### IS SEA-SICKNESS A MYTH?

It is popularly believed that all landsmen, speaking generally, cannot hope to travel by sea without being seriously sick. This belief is based no doubt upon the fate which usually befalls those who take an occasional trip across the Channel. Formerly the Channel steamers were so small and ill-found that passengers had not only no comfort, but more frequently than not a good wetting, or even several wettings if the sea was fresh. On some routes this is still the case, and anyone who can cross from Newhaven to Dieppe when the sea is moderately fresh without being the worse for it, may still claim to be a good sailor. The new steamers of the *Victoria* type, which now run from Dover and Folkestone to Calais and Boulogne, as well as those which ply from Harwich, and even from Queenborough, to Flushing or Antwerp, are so well constructed and found that the average traveller rarely feels much if any discomfort in the course of his journey. What he does feel, despite the comforts and excellences of the steamers, is no doubt due to the "choppy" character of the sea in the Channel. Even Americans, who have crossed the Atlantic many times without illness, declare that the Channel steamer is more often than not more than they can stand. In these circumstances it would be wrong to conclude that one was a bad sailor because of the discomfort felt in crossing from Dover to Calais or Harwich to Flushing, much less from Newhaven to Dieppe or from Folkestone to Ostend. In the two latter cases the boats would probably upset an admiral, and no judgment can be formed of the sailing powers of anyone from the results of such a voyage. Choppy seas and small and inadequate steamers would be quite enough to upset the best seaman, especially if he came on board in a condition of health ill suited to face a good tossing on troubled waters. Another popular error is the belief that the sea, as a rule, is very rough, and that the further a vessel goes from the land the greater is the probability that the weather will be bad and unendurable. Of course heavy seas will at times be encountered—they are, by-the-by, often preferable to the "choppy" Channel—but there is as good, and probably more fine weather at sea during each twelve months than on land. Then again, the cruises which were set out in a previous article are so arranged as to give the traveller a frequent rest on shore, whilst the time of the year selected for each is so arranged as to give the maximum probability that fine weather will be experienced throughout. Let us illustrate this by an example. The Baltic cruise commenced on August 30th and terminated on September 28th. From the former date to September 25th no rough seas were encountered, and the weather, with the exception of a few hours on one day, was beautifully fine, temperate and congenial. Whilst friends at home were grumbling at the horrid weather, so wet, cold, and trying to everybody, those who were cruising in the Baltic and North Seas had the brightest and best of days and the most balmy and brilliant evenings. On the few days and nights when the temperature was lower, a little extra clothing sufficed to render it pleasant rather than the reverse. Having thus cleared away two prevailing errors in reference to sea travel, we pass on to consider whether sea-sickness is a necessary evil which most people who take a voyage for pleasure must make up their minds to submit to. Of course there are some persons with so little self-restraint, or so constituted that they begin to be ill as the train which takes them to the steamer leaves the London terminus. We have seen passengers going to Ostend or Boulogne give up the ghost at Victoria, and before Herne Hill was passed they had assumed a yellow aspect, had draped themselves in the *déshabille* which such passengers by sea usually adopt, and were extended on the seat of the carriage, or were already reclining upon pillows, after taking one or more doses of the medicines which they had seen advertised as preventitives, or been recommended by friends to swallow. Another class of persons believes in taking alcohol on such occasions, not wisely but too well, bottled stout being a beverage specially favoured by many such. No earthly power could save such people from being sea-sick, and many of them manage to work themselves into a state of biliousness before the steamer starts, which could but end in one way, whether they went to sea or remained at home. Such people work very hard to make themselves miserably ill whenever they have to cross the seas, and who shall wish to deprive them of the melancholy satisfaction which is, or ought to be theirs, when they succeed in the attempt?

By the ordinary traveller, however, who really possesses common sense and self-respect, sea-sickness need rarely, if ever, be experienced, providing he attends to a few simple details, which are as necessary to comfort in making long railway journeys as they are on sea-going vessels. First, the general health should be attended to a few days before leaving home, and the system should be overhauled as systematically as the clothes and books selected for the journey. Many people make the mistake of adopting some special plan of living when at sea, whereas a moderate eater with any sense will find it far better to live as he does on shore, and not to risk an upset to his digestion by trying all sorts of things he would not touch at home. Moderation in all things, and especially in food and alcohol, are no doubt desirable, but the invigorating influence of sea and air must be allowed to have a free hand, if health and comfort are desired. A variety of clothing is also a *sine qua non*. The temperate character of our climate leads many people to pay little, if any, attention to the kind of clothing that is suitable to different seasons, and it is not uncommon to hear a man boast that he makes no difference, but wears the same clothing all the year round. Much avoidable discomfort is experienced by fools, as Solomon and later sages have proved to demonstration, and amongst the number those here referred to must be included. At sea the temperature often varies ten degrees at least in a few hours, and warm as well as light clothing should be taken on all voyages in sufficient quantity. To be too warm is unpleasant; to be too cold, or to woo the king of causes of disease, chill, by wearing improper clothing, is little short of madness. Care should also be taken to prevent getting wet. Everybody on a steamer likes as much air as possible, but it is not sufficiently realised that there are very many unexpected causes which may give a passenger a soaking without warning and when least expected. A good mackintosh and a waterproof rug and hat are therefore indispensable. On a short voyage, in a small and crowded vessel, the risks of a wetting are manifold, and the present writer remembers to this day with horror the misery experienced on a journey from Dieppe to Newhaven with a warm sun overhead, when he was drenched to the skin three times by the waves which broke over the vessel.

Finally, in a voyage of any duration, exercise and occupation are essentials to be insisted upon. It is found in the large ocean-going steamers that the stewards would very frequently be sea-sick if they were not kept occupied and at work. When sickness has commenced this holds good, and the man who will courageously fight against it by taking exercise, with assistance when necessary, will soon trample down his foe. Walking for an hour a day at a good pace is one way of securing this essential to health on board ship. The observant will notice that the seamen are never allowed to be idle. So long as daylight lasts they are kept at work, and in no other way would they remain fit for duty. So also among the passengers, exercise and occupation are as essential to health, as they are certain to remove or prevent sea-sickness. Sea quoits are one unfailing source of exercise and amusement. A man may keep himself warm in this way on the coldest day, whilst the incentive which a match between two average players at varying distances will excite cannot fail to relieve the mind from anxiety as to the effect the motion of the vessel will have on the individual. Music, society, charades, theatricals, dancing, sea archery, sea cricket, chess, whist, anything and everything in fact which diverts without wearying the mind should be encouraged and had resort to. Who that sits on deck watching the motion of the vessel, and wondering "When, oh, when shall I be ill?" can reasonably expect to remain well? Let those who doubt the force of our contentions try a simple experiment. Let them attach a ball to a piece of string, suspend it from the ceiling at the foot of the bed, and when they wake in the morning feeling rather squeamish and out of sorts let them set the ball moving from side to side across the bed. If they will then watch the motions of the ball with absorbed attention, like the benighted passenger referred to, they will find it necessary before long to send for the steward and the basin, or their substitutes, although they are reclining safely in their own bed at home. With resolution, courage, good general health, ordinary diet, proper clothing, freedom from wet, exercise and occupation, life at sea will be found as healthy, and, in many respects, far more enjoyable than life ashore, and sea-sickness as a necessary and common complaint will be proved by the majority of mankind to be a myth. We refrain from giving a list of preventives to sea-sickness, that is medical antidotes, because though useful they are seldom or never necessary for the health of those who take a voyage in vacation. Amongst them cocaine seems to stand first, whilst several of the elegant preparations of Messrs. Burroughs, Welcome, and Co. are highly spoken of by the faculty.

*(To be continued.)*

